# In-vitro endotracheal tube cuff pressure decreases following transport via helicopter: implications for medical evacuation of military working dogs

**DOI:** 10.1186/s12917-025-05146-4

**Published:** 2025-12-15

**Authors:** Samuel M. Tucker, Margaret-Mary McEwen, Shane J. Andrews, Lysa P. Posner

**Affiliations:** 1 US Army Veterinary Corps, APO, AE USA; 2 VetPrac Continuing Education Pty Ltd, Ipswich, QLD Australia; 3 North Carolina State University College of Veterinary Medicine, Raleigh, NC USA

**Keywords:** Endotracheal tubes, Altitude, Military working dog, Cuff pressure, MEDEVAC

## Abstract

**Background:**

Altitude change impacts endotracheal tube cuff pressure (ETTCP) in human patients that are medically evacuated (MEDEVAC) via flight. While Military Working Dogs (MWD) are routinely transported via helicopter during evacuations for life threatening injuries, the effects of altitude on ETTCP have not been evaluated in veterinary medicine. This study aimed to evaluate if changes in altitude following transport in a helicopter impacts ETTCP in veterinary-grade endotracheal tubes (ETT) as seen in intubated human patients that are MEDEVAC via flight.

**Methods:**

In an observational in-vitro study, 14 new, 11 mm polyvinyl chloride veterinary ETT were evenly divided into two groups (G1 and G2). All ETT were inflated to an initial ETTCP of 30 cmH_2_O at ground-level prior to the flight using one handheld manometer. All ETT were then transported together in a single helicopter flight for 1 h at varying altitudes up to 1022 m above sea level. ETTCP was measured via the same handheld manometer. In G1, ETTCP was measured twice, at inflation and following landing. In G2, ETTCP was measured at inflation and every 15 min following takeoff until landing. Altitude, barometric pressure, temperature (in and outside of the aircraft), and humidity were recorded. Groups were compared with a paired t-test (G1) and an ANOVA (f-test) followed by pairwise t-tests (G2) with significance set at *p* < 0.05.

**Results:**

There was a 10 cmH_2_O or more decrease in ETTCP between the initial and final measurements in 71% and 100% ETT in G1 and G2, respectively. In G2, decreased ETTCP values became clinically relevant and statistically significant at 30 min post-takeoff.

**Conclusion:**

ETTCP fell below 20 cmH_2_O in 86% of ETT tested. During and following MEDEVAC in intubated canine patients, ETTCP should be reevaluated and adjusted.

**Supplementary Information:**

The online version contains supplementary material available at 10.1186/s12917-025-05146-4.

## Introduction

Endotracheal intubation is performed in anesthetized and/or critical patients to ensure a sealed, protected airway and facilitate the delivery of oxygen, intermittent positive pressure ventilation (IPPV), and/or inhalant anesthetic gases [1–4]. A sealed airway is created through the inflation of the endotracheal tubes’ (ETT) cuff (ETTC) with a recommended ETTC pressure (ETTCP) range of 20–30 cmH_2_O [1, 3, 5–9]. Of note, this range is based upon work in animal models and human medical guidelines as no true veterinary specific ETTCP range has been described [10]. Overinflation of an ETTC can result in excessive pressure placed onto the tracheal mucosa and surrounding anatomy that disrupts normal blood flow [1, 9, 11]. ETTCP exceeding 30 cmH_2_O is associated with reduced mucosal tissue perfusion and ETTCP above 50 cmH_2_O resulted in complete obstruction or rupture of the trachea [9, 11–15]. Conversely, ETTCP below 20 cmH_2_O can result in unsealed airways and aspiration of oropharyngeal contents [16, 17].

Human patients are often intubated prior to medical evacuations (MEDEVAC) using aircraft (helicopters or fixed-wing aircraft) [2, 12, 15, 18]. As described by Boyle’s Law, at a constant temperature, pressure (P) and volume (V) are inversely proportional (P $\:\propto\:$ 1/V). Thus, as altitude increases and atmospheric pressure decreases, the gas volume trapped within inflated ETT can expand, increasing the ETTCP and negatively impact tracheal mucosal health [2, 12, 15, 19]. In human medicine, multiple studies have demonstrated that increased ETTCP is a commonly encountered complication following flights at high altitudes [12, 15, 20–22]. No readily identifiable risk factors for elevated ETTCP, for both hospitalized and transported patients, have been described in regard to patient age, sex, ETT size, skill level of individual intubating, or time frame between intubation and ETTCP measurement [2, 23].

Military Working Dogs (MWD) also undergo MEDEVAC for life-threatening injuries or illnesses. Despite the multiple studies exploring the relationship between altitude and ETTCP in human patients, there is only one report on veterinary patients. The single report was a retrospective study evaluating cases of MWD undergoing MEDEVAC with minimal discussion on the management of ETTCP due to altitude changes [24]. Another case report (albeit at ground-level) in a canine described tracheal necrosis resulting from overinflation of the ETTC (alongside multiple patient repositioning movements) but the exact ETTCP was not recorded [11]. To the authors’ knowledge, there are no systematic investigations of the effects of altitude on ETTCP in veterinary-grade ETT during helicopter flights. Given that MWD can be critical patients who frequently require MEDEVAC and the likelihood they suffer adverse consequences due to changes in ETTCP from increasing and decreasing altitudes (e.g. mucosal injury and/or aspiration due to inadequate ETTC seal), investigating the effects of altitude changes on veterinary-grade ETT is needed.

The aim of this study was to evaluate if changes in altitude alter ETTCP in veterinary-grade ETT. It was hypothesized that as altitude increased, the ETTCP would similarly increase in accordance with the principle of Boyle’s Law, resulting in ETTCP above the recommended 20–30 cmH_2_O during flight.

## Materials and methods

Fourteen, new, polyvinyl chloride 11 mm ETT (Dee Veterinary Products, Miami Gardens Florida, USA 33169) were divided into two groups: Group 1 (G1) and Group 2 (G2) (*n* = 7/ETT group). All ETT were transported together in an unpressurized cabin for an approximate 1 h flight time in a United States Army UH-60 M Black Hawk helicopter at varying altitude levels (up to 1022 m above sea level) at the Grafenwoehr Airfield, Germany (approximately 412 m in elevation above sea level). On the date of data collection, air temperature at ground-level ranged from 4.4 ℃ to 6.1 ℃. All ETT were manually inflated to 30 cmH_2_O within the helicopter approximately 54 min prior to takeoff during the preflight checks using a Posey Cufflator Manometer™ (TIDI Products, LLC; Neenah, WI, USA 54956). The ETTC was inflated by connecting the pilot tube of the ETT to the manometer and manually squeezing the handle to gradually inflate the ETTC. Measurements of ETTCP were obtained by gently connecting the same pilot tube to the adapter on the manometer. Air pressure in the ETTC moved the manometer gauge to a correlating number (values in cmH_2_O).

A preliminary study at ground-level (411 m) in a climate-controlled setting (temperature range: 22.5 ℃ − 22.9 ℃) using the same ETT utilized in this study and Posey manometer was performed to assess the feasibility of sampling times and equipment. Seven ETT were inflated to 30 cmH_2_O with ETTCP measurements obtained every 5 min for 1 h as previously described. Over the course of 1 h, a steady decrease in ETTCP was observed to an average final ETTCP of 11.2 cmH_2_O. Given that all ETT were new, and that deflation was observed in all seven tested inflated ETT, a defect in the ETT was not suspected though lack of cuff integrity in all seven ETT cannot be ruled out. Maximum temperature change within the room was 0.4 ℃ thereby eliminating environmental variables as a factor. The manometer itself is routinely serviced and utilized by the Landstuhl Regional Medical Center’s Anesthesiology Department and so, an equipment malfunction was also less likely. The observed deflation was attributed to possible air column sampling from the manometer following attachment to the ETT pilot line every 5 min during measurement attempts. This assumption is supported by a previous in-vitro study in which connection of a manometer to the pilot balloon resulted in decreased ETTCP in 78.1% of cases [25]. Air sampling from manometers has been attributed to decreased ETTCP and under-inflation in other studies as well [26, 27]. Therefore, it was elected to sample the ETT every 15 min in the experimental group with multiple sampling times to decrease this air loss.

Group 1 (G1) ET had ETTCP measured twice; once at initial cuff inflation and once following landing. In Group 2 (G2), ETTCP was measured at cuff inflation and every 15 min after takeoff and immediately following landing. G1 was only measured once after initial inflation in order to compare with G2 in which multiple samplings were performed over the course of the flight.

Altitude (measured in feet above ground level with estimated elevation added and reported in meters), barometric pressure (mmHg), and temperature (outside of the aircraft) (℃) were measured via onboard equipment on the helicopter. Temperature (within the aircraft) (℃) and humidity (%) were measured via portable digital thermometers (ThermoPro TP49W-3 Digital Mini Thermo-Hygrometer Thermometer, Dusseldorf, Germany 40219). At takeoff, a timer was started to determine data collection at the described 15-minute intervals for G2. Final measurements of ETTCP in both G1 and G2 occurred at landing approximately 66 min after takeoff.

### Statistical analysis

Data analysis was performed using R (R version 4.4.1). The assumption of data normality was tested using the Shapiro-Wilk test with *p >* 0.05. Therefore, parametric tests (t-test and F-test) were utilized for further analysis. For G1, changes in ETTCP were analyzed with a paired t-test. For G2, changes in ETTCP were analyzed with a one-way ANOVA (F-test) followed by pairwise t-tests for each pair of measure points at 15, 30, 45, and 65 min compared to the initial baseline measurement. The calculated *p* values were adjusted using the Tukey method for family-wise test errors. A separate t-test was performed comparing the final ETTCP of G1 with the final ETTCP of G2. Statistical significance was set at *p* < 0.05.

## Results

**Fig. 1:**
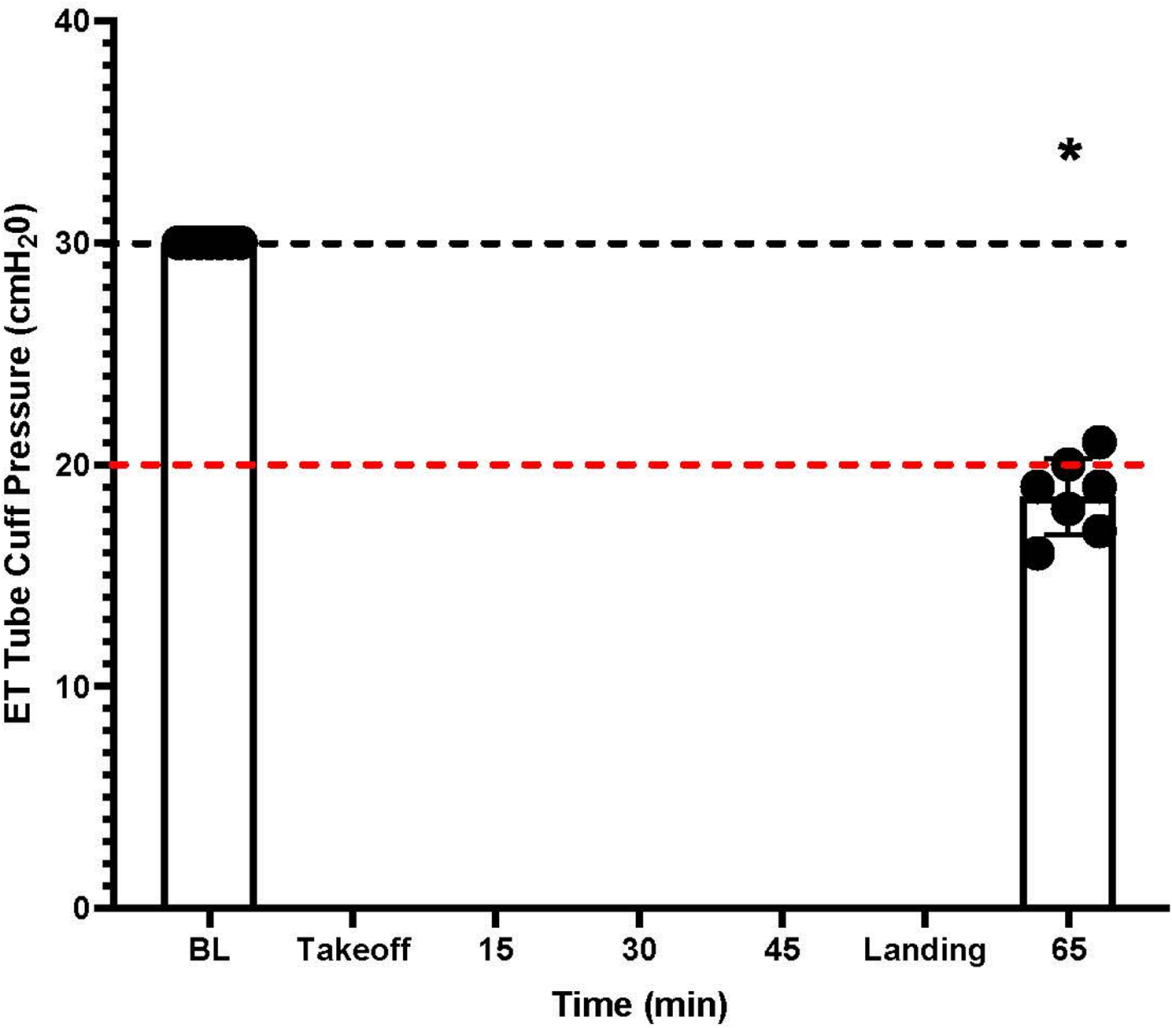
Results of group 1 (G1) endotracheal tube cuff pressure (ETTCP) over the course of an approximately 1 h helicopter flight. The measured ETTCP of seven endotracheal tubes (ETT) (Black circle = individual ETT) from a baseline (BL) inflation of 30 cmH_2_O (black-dashed line) are displayed. The x-axis represents the time in minutes from the initial inflation of the ETT to landing at 66-min post-takeoff, while the y-axis represents the ETTCP in cmH_2_O. 86% of ETT were below the recommend minimum ETTCP of 20 cmH_2_O (red-dashed line) after landing. Standard deviation of mean from ETTCP measurements are displayed as brackets. Collective measurement that is significantly different compared to BL is denoted (*)

Mean (± SD) ETTCP in G1 at baseline and following landing was 30 (± 0) and 18.6 (± 1.3) cmH_2_O, respectively. There was a significant decrease in ETTCP (*p* = 0.000002) between the baseline and final ETTCP measurements (Fig. 1).

**Fig. 2:**
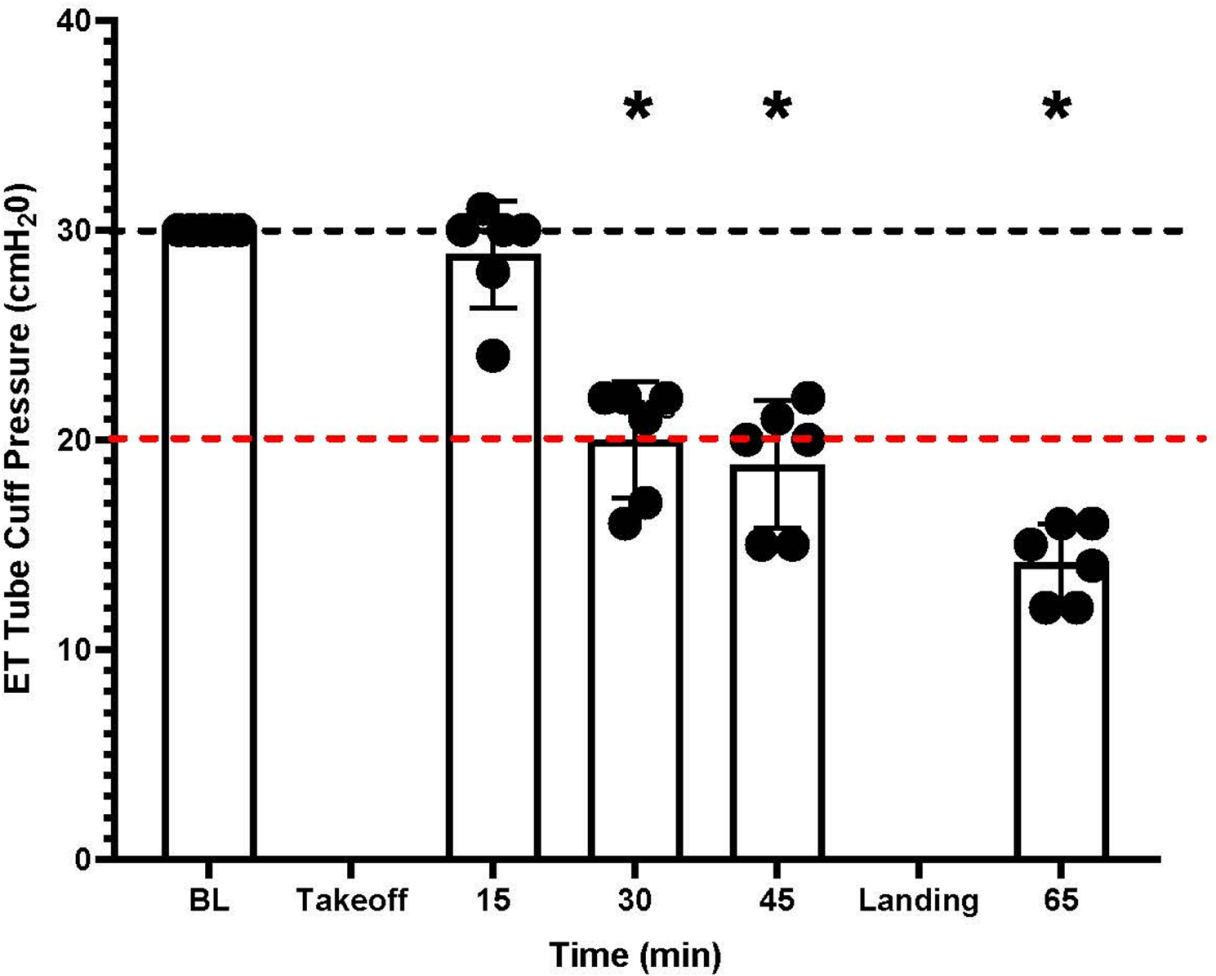
Results of group 2 (G2) endotracheal tube cuff pressure (ETTCP) over the course of an approximately 1 h helicopter flight with measurements collected every 15 min following takeoff. The measured ETTCP of seven endotracheal tubes (ETT) (Black circle = individual ETT) from a baseline (BL) inflation of 30 cmH_2_O (black-dashed line) are displayed. The x-axis represents the time in minutes from the initial inflation of the ETT to landing at 66-min post-takeoff, while the y-axis represents the ETTCP in cmH_2_O. Standard deviation of mean from ETTCP measurements are displayed as brackets. All values were below the recommend minimum ETTCP of 20 cmH_2_O (red-dashed line) after landing. Time intervals that are significantly different compared to BL are denoted (*)

Mean (± SD) ETTCP in G2 at baseline was 30 (± 0) cmH_2_O. Mean (± SD) ETTCP in G2 at 15, 30, and 45 min in flight and following landing was 28.7 (± 1.8), 20 (± 2), and 19 (± 2.3) and 14.1 (± 1.3) cmH_2_O, respectively. There was a significant decrease in ETTCP at 30 (*p* = 0.00), 45 (*p* = 0.00) and following landing (*p* = 0.00) time points compared with baseline (Fig. 2).

Mean (± SD) final ETTCP of G1 (18.6 (± 1.3) cmH_2_O) was significantly greater than mean (± SD) final ETTCP of G2 (14.1 (± 1.3) cmH_2_O) (*p* = 0.0004).

Over the course of an approximately 1 h flight time, there was a 10 cmH_2_O or more decrease in ETTCP between the initial and final measurements in 71% and 100% ETT in G1 and G2, respectively.

Maximum and minimum recorded altitude above sea level in flight were 1022 and 747 m, respectively. These correspond to altitude changes of 613 and 338 m, respectively, when compared to the starting elevation of 412 m above sea level. The maximum recorded elevation difference (579 m) occurred between the 45-min timepoint and landing. The maximum flight altitude was reached within 30 min of the flight (1022 m). Maximum temperature changes inside and outside the helicopter were 6.7 ℃ (10.5–17.2 ℃) and 4 ℃ (2–6 ℃), respectively. The maximum and minimum recorded barometric pressures during flight were 771.1 mmHg and 762 mmHg, respectively.

## Discussion

In this study, there was a statistically significant decrease in ETTCP from initial ETTC inflation prior to takeoff compared to those at landing following a 1 h flight in a helicopter. This was also clinically relevant as ETTCP fell below the recommended range of 20–30 cmH_2_O. As set forth by Boyle’s Law, at a constant temperature, as atmospheric pressure decreases with increasing altitude, volume increases. Therefore, with higher altitudes, the gas volume within the cuff of an inflated ETT should subsequently increase. While there are many reports regarding changes in ETTCP in human MEDEVAC patients, the reports are not consistent in reporting altitude, barometric pressure, temperature, or duration of flight, so, the ability to generalize expectations is limited. Most reports are concerned with and have described increases in ETTCP following increased elevations [2, 12, 15, 18, 20], however, a decrease in ETTCP and the need for cuff inflation has been reported with one study, albeit in a pressurized cabin, documenting up to 85% of ETTC that required re-inflation upon landing [15]. In contrast to our hypothesis, the present study did not demonstrate increasing ETTCP with an increase in altitude up to 613 m. Possible causes for the lack of increase in ETTCP include moderate altitude elevation on this flight, the rapidity of altitude changes, the duration of flight, and the type of ETT. While the lack of increase in ETTCP was most likely due to moderate elevation changes (barometric pressure changed from only 771 mmHg to 762 mmHg), a study in humans reported that gradually increasing ETTCP were observed in a flight to a mean altitude of 688 m in which 98% of ETTCP exceeded 30 cmH_2_O, 72% exceeded 50 cmH_2_O, and 20% were greater than 80 cmH_2_O [20]. Elevated ETTCP have similarly been observed in increased elevations of only approximately 152 m where ETTCP was greater than 30 cmH_2_O in 64.7% of patients [2]. Interestingly, less clinically relevant changes have been reported at similar altitudes changes of 305 to 914 m where mean ETTCP was approximately 36 cmH_2_O [21].

Another possible explanation for the lack of observed increased ETTCP is that ETTCP might have been truly elevated at some timepoint during the flight but not at one of the 15-min sampling time points due to the nonlinear ascent and descent of the flying helicopter. Large increases in ETTCP at certain elevations might have been missed if they occurred outside of the measuring time points. To the authors’ knowledge, the rapidity of ascent and descent along with the duration at certain elevations and the resulting effect on ETTCP has not been studied. In this study, the flight decisions were made by the pilots and ascent and descent were not controlled, so it is possible that the rapidity of altitude change affected the results.

The larger size ETT used in this study could also explain the absent increase in ETTCP following the flight. As ETT size increases, it is possible that the pressure increase becomes less observable depending upon the altitude. In one human study evaluating ETTCP up to 2,400 m, size 6.5 mm ETT had an increase in ETTCP of 3.0 cmH_2_O per 100 m compared to size 7.5 mm ETT which had an increase in ETTCP of 2.1 cmH_2_O per 100 m [12]. In the current study, only one size of ETT was used. While there may be differences observed across different sized ETT given the diversity of dog breeds and sizes, the size included in this study was selected to represent those likely to be used in military working dog breeds (i.e. German Shepherds and Belgian Malinois).

The decrease in ETTCP was consistent within and between the groups. Potential causes for the decrease include losses due to sampling with the manometer, changes in temperature, intrinsic properties of the cuff or a cuff defect. Since only one manometer was used, it had to be attached to each ETTC pilot valve at each sampling time. Air pressure from the ETTC is directed into the manometer with each sampling which could contribute to small losses of air from the cuff. This hypothesis is further supported by a greater observed loss of ETTCP from the ETT in G2 compared with G1. The manufacturer did not report the volume needed to fill the tubing of the manometer, so the exact amount lost to this sampling is unknown, but considering the G1 final value only had one additional measure from the initial measurement, it is likely that actual loss occurred rather than from sampling losses alone which would account for a >10 cmH_2_O difference. However, as previously discussed, decreased ETTCP has been reported as a consequence of repeatedly connecting a manometer to the pilot balloon [25–27]. In one study, ETT set to an ETTCP of 20 cmH_2_O experienced an initial decrease to an ETTCP of 13.6 cmH_2_O (range: 4.6–23.0) following the first measurement with a manometer [25, 27]. While not the goal of the study, another retrospective found that 85% of ETT in intubated patients transported via medical aircraft had to be readjusted to a higher ETTCP following landing [15]. Given the retrospective nature of that study, it is difficult to determine if sampling, other variables, or true influence from descent affected the ETTCP.

Changes in the temperature of the gas within the ETTC is another variable that could have affected ETTCP. The air temperature in the cabin of the helicopter ranged from 10.5 to 17.2 ℃ with a decrease of 6.7 ℃, which could have contributed to a decrease in ETTCP. A patient’s body temperature affects ETTCP as significant differences have been observed between the ETTCP of uncontained ET kept at room temperature and those maintained at body temperature [17]. Thus, it is possible the in-vitro nature of the experience exacerbated this change since the ETT was not being warmed by the body temperature of a patient. It is also likely that the temperature of the gas within the ETTC changes more rapidly than it would in a live patient. Further studies are needed to correlate altitude, temperature, and ETTC volume and pressure.

While there are different styles and brands of ETT, a single batch of polyvinyl chloride ETT were used in this study due to convenience. It is possible that this batch of ETT from the same manufacturing lot could have been defective and resulted in the increased ETTCP observed. Another possible explanation for the decreased ETTCP could be due to the properties of the cuff. Another study using polyvinyl chloride ETT described the elastic nature of the ETT (compared to a balloon) as a potential cause for the decrease ETTCP [17]. Future studies exploring changes in ETTCP following altitude changes across different sizes, types, and brands of ETT could determine if altitude universally affects all ETT regardless of ETT material and size.

An inappropriately filled ETTC can result in clinically relevant sequela. Overinflation can result in mechanical/ischemic trauma to the trachea or tracheal mucosa whereas underinflation can result in an unsealed airway which can impede positive pressure ventilation and/or allow aspiration of gastrointestinal contents. While underinflation can be detected by providing positive pressure and measuring the airway pressure via a manometer connected to the ETT, there are few objective ways to determine overinflation without assessing ETTCP. It is surprising that in human and veterinary medicine, filling the ETTC is most commonly performed without assessing ETTCP but rather ETTC are inflated via syringes with ETTCP estimated by either pilot-balloon palpation (PBP) or minimal occlusion volume (MOV) even with evidence that human medical providers have been found to be unable to readily detect excessive ETTCP [3, 28]. Interestingly, a study in human intensive care units (ICU) reported that 75% of ICU never routinely checked ETTCP and of the ETTCP that were measured, 62% exceeded recommended values [29]. In veterinary medicine, a survey found that less than one-third of respondents even measured ETCP [10]. Based on Boyle’s Law, patients with overinflated ETTC would be at a greater risk for barotrauma to the trachea with changes in altitude. Particularly in the scenario of MEDEVAC with expected changes in altitude, the use of a ETTCP manometer should be considered [4, 15, 20, 28].

Since barotrauma from an overinflated ETTC or expansion of the ETTC at altitude presents real risks to patients, several studies have evaluated alternatives to air for inflation of the ETTC in patients who will be transported in aircraft. Inflating the ETTC with saline rather than air has been suggested as a mitigation for the effects of elevated ETTCP due to increasing altitudes [12, 24, 30, 31], however, other studies have found that ETTCP were still elevated during flight and upon landing at sea level [30]. Furthermore, other studies have found that a saline-filled ETTC is not needed under altitudes of 914 m [21] whereas others recommend against using saline for inflation completely [32]. It was not possible to evaluate saline as an alternative inflation method in this study as the manufacturer guidelines for the manometer utilized recommended against it due to risk of inaccurate measurements.

In contrast to using air or saline for cuff-inflation, the use of automatic cuff pressure controllers are designed to maintain consistent ETTCP despite increases in altitude in simulated flights as the actual pressure of the cuff is controlled rather than relying upon an injected volume (air, liquid) [19, 32]. Yet, the use of a cuff pressure controller only reduced ETTCP to 36 mmHg (48.9 cmH_2_O) in a flight up to approximately 2,400 m and ETTCP fell below 15 mmHg (20.4 cmH_2_O) upon landing [30]. This study was limited in comparison of different cuff-inflation techniques as only one type of manometer was available. Therefore, comparison of different equipment on ETTCP is warranted.

The importance of appropriate inflation of ETTC is magnified in the MEDEVAC of an MWD. These patients are critically ill and often transported from hostile settings. Additionally, these patients are at a greater risk for aspiration as they may not have been fasted nor administered anti-nausea or anti-emetics prior to anesthesia and transport. They are also subject to increased movements both by moving into and out of the helicopter, but also from the movement of the helicopter itself during the flight. Furthermore, most MWD that undergo MEDEVAC do so with limited veterinary support [24]. While MWD handlers are routinely present during MEDEVAC, in a retrospective study, only 8% and 11% of cases had a veterinarian or veterinary technician as part of the medical care team, respectively [24]. Therefore, even though MWD handlers are trained in life-saving techniques for their canines, most medical professionals readily available in these circumstances would be human medical professionals. As previously mentioned, human healthcare providers have been found to be unaware of recommended ETTCP ranges in humans, provide inappropriate ETTCP to patients, and not routinely monitor ETTCP [3, 5, 23, 28, 29, 33]. Based upon their familiarization with ETCP, and available equipment, it is possible that MWD may have inappropriately inflated ETT which could complicate already critical patients depending upon the depth of experience the human care provider has with MWD emergency medicine techniques.

This study had several limitations. First, was the in-vitro nature of this study. In one comparative study, a model suggested that ETT that were confined within a simulated trachea (plastic tubing) would develop an ETTCP of 50 cmH_2_O at only 400 m compared to unconfined ETT that did not reach those pressures until 600 m [12]. Future studies should consider the inclusion of cadavers, which were not feasible in this study, or other modes of simulating a trachea to evaluate confinement and different temperatures on ETTCP. This study was also limited in availability of manometers. Even though the single manometer used was serviced by a human medical hospital, the potential for measurement bias exists. Ideally, all ETT would have had a single dedicated manometer (which would have helped reduce further air sampling from connecting and re-connecting a manometer during each measurement), but this was not possible in the current study. There was approximately 1 h from initial inflation of the ETTC to flight takeoff. Preferably, the ETT would have been inflated just prior to takeoff but this accurately reflects the clinical patient as they would have been intubated with the ETTC inflated prior to loading and securement within an aircraft. Of note, despite this long interval from inflation to takeoff, four ETT in G2 had ETCP >30 cmH_2_O at the first measurement 15 min after takeoff. Finally, while a feasibility study was performed prior to the actual study assessing the effects of manometry on sampling, these samples were recorded every 5 min over the course of an hour. It would have been beneficial to replicate this at ground-level with the same ETT and manometer and measure ETTCP every 15 min to compare with the ETT transported.

## Conclusion and clinical relevance

Following a 1 h helicopter flight, ETTCP decreased below the recommended range of 20–30 cmH_2_O in one brand of size 11 mm polyvinyl chloride ETT. These changes in ETTCP have the potential to increase risk of aspiration in already critical MWD undergoing MEDEVAC. The use of cuff manometry is recommended to continually assess and correct ETTCP in MEDEVAC canine patients.

## Supplementary Information

Supplementary Material 1.

## Data Availability

All data on the measured ETTCP during the flight that support the findings of this study are included within this paper and its Supplementary Information files.
